# Insights into Blue Whale (*Balaenoptera musculus* L.) Population Movements in the Galapagos Archipelago and Southeast Pacific

**DOI:** 10.3390/ani14182707

**Published:** 2024-09-18

**Authors:** Hector M. Guzman, Rocío M. Estévez, Stefanie Kaiser

**Affiliations:** 1 Naos Marine Laboratory, Smithsonian Tropical Research Institute, Panama City P.O. Box 0843-03092, Panama; guzmanh@si.edu (H.M.G.); estevezr@si.edu (R.M.E.); 2 Senckenberg Research Institute and Natural History Museum, 60325 Frankfurt am Main, Germany

**Keywords:** Southeastern Pacific, satellite tracking, thermal dome, hidden Markov model (HMM), state–space model, migratory behavior, Carnegie Ridge, Nazca Ridge, wintering and calving areas

## Abstract

**Simple Summary:**

The Galapagos Marine Reserve supports cetaceans such as blue whales by providing essential foraging areas and resting areas. From 2021 to 2023, a study utilized satellite tags on 16 blue whales to monitor their movements and gain insights into their interactions with the marine environment. This involved the identification of their feeding grounds, indicated by chlorophyll levels, surface water temperature (SST), and ocean productivity. The study revealed that blue whales prefer areas with presumed increased prey abundance, cooler water temperatures, and specific underwater features such as ridges that likely enhance favored food sources. Most of the tagged whales remained in the Galapagos, particularly near Isabela Island, where there is an elevated risk of collisions with boats due to increased marine traffic. Some whales ventured into Ecuador’s waters, and one even traveled to Peru. In 2023, an intense El Niño event led to changes in water temperatures and food availability, significantly impacting whale habitats. This research contributes to efforts to protect whales from ship collisions and understand and adapt to changes in their migration patterns due to climate variations.

**Abstract:**

The Galapagos Marine Reserve is vital for cetaceans, serving as both a stopover and residency site. However, blue whales, occasionally sighted here, exhibit poorly understood migratory behavior within the Galapagos and the broader Eastern Tropical Pacific. This study, the first to satellite tag blue whales in the Galapagos (16 tagged between 2021 and 2023), explored their behavior in relation to environmental variables like chlorophyll-a concentration, sea surface temperature (SST), and productivity. Key findings show a strong correlation between foraging behavior, high chlorophyll-a levels, productivity, and lower SSTs, indicating a preference for food-rich areas. Additionally, there is a notable association with geomorphic features like ridges, which potentially enhance food abundance. Most tagged whales stayed near the Galapagos archipelago, with higher concentrations observed around Isabela Island, which is increasingly frequented by tourist vessels, posing heightened ship strike risks. Some whales ventured into Ecuador’s exclusive economic zone, while one migrated southward to Peru. The strong 2023 El Niño–Southern Oscillation event led to SST and primary production changes, likely impacting whale resource availability. Our study provides crucial insights into blue whale habitat utilization, informing adaptive management strategies to mitigate ship strike risks and address altered migration routes due to climate-driven environmental shifts.

## 1. Introduction

Escalating and cumulative impacts of human activities on oceanic ecosystems, such as overexploitation, habitat destruction, pollution, and climate change, pose considerable threats to marine biodiversity, leading to notable population declines and pushing numerous species toward extinction [1,2]. As awareness of the importance of healthy oceans grows, protecting biodiversity and its critical ecosystem services, such as food provisioning and carbon sequestration, has become essential for maintaining ecosystem stability and resilience [3,4,5]. The effective conservation of marine biodiversity requires mapping and understanding species distributions and the environmental factors affecting them. However, most marine species still require further study [6]. Remarkably, this trend extends even to the largest marine species, the blue whale (*Balaenoptera musculus* L.), whose migratory behavior across vast oceanic spaces complicates efforts to accurately assess population numbers and distributions [7].

Currently, *B. musculus* is classified as endangered according to the International Union for Conservation of Nature (IUCN) Red List of Threatened Species [8]. Decades of intensive commercial whaling during the 20th century decimated their populations, especially in the Southern Ocean, pushing them to the brink of extinction [9,10]. Although some regions show signs of recovery, populations remain well below pre-whaling levels [9,10,11]. Taxonomic uncertainties, distribution challenges, and limited data on seasonal movements due to variable monitoring efforts impede global and regional population assessments [10,12,13,14].

Worldwide, two to four subspecies within *B. musculus* have been distinguished morphologically, genetically, and geographically [15,16]. In the Southern Hemisphere, these include the Antarctic blue whale, *B. m. intermedia*, and the Pygmy blue whale, *B. m. brevicauda* [17]. In the Northern Hemisphere, *B. m. indica* occupies the North Indian Ocean, and *B. m. musculus* is found in the North Atlantic and North Pacific oceans [18,19]. Taxonomic uncertainty persists for *B. m. brevicauda* and *B. m. indica*, with differentiation primarily based on song types [18,19]. In the Eastern South Pacific, a potentially distinct population or subspecies known as the Chilean blue whale has been suggested to exist, though formal recognition is pending [10,14,20]. These subspecies are further divided into populations based on migration routes, regions, and song types, with vocalizations identifying eleven distinct vocal populations globally [12,21,22,23,24,25,26,27].

The conventional understanding is that blue whales alternate between high-latitude nutrient-rich waters to feed almost exclusively on euphausiids (krill) during the summer and low-latitude breeding grounds during the winter [28,29,30]. However, sightings, captures, and acoustic evidence suggest a year-round presence at various latitudes, challenging this paradigm [31,32,33,34]. Deviations from this pattern can lead to more opportunities for different populations to co-occur in the same location [26,35,36].

The Eastern Tropical Pacific (ETP), located between the subtropical gyres of the North and South Pacific, is a shared wintering area for blue whales from the Northeast Pacific (NEP) and Southeast Pacific (SEP) populations [14]. Whales primarily aggregate around Baja California, the Thermal Dome (previously described as the Costa Rica Dome), offshore Peru, and the Galapagos archipelago [14,29]. Comprehensive data on movements and habitat use within the ETP are limited due to the rarity of sightings and challenges in studying these remote areas [36]. The Thermal Dome is believed to be a breeding area for NEP whales, while the Galapagos Marine Reserve is a significant wintering and potential breeding area for SEP whales [23,29,31,37,38]. There is, however, evidence of a year-round blue whale population in the Galapagos [35], as well as the co-occurrence of NEP and SEP migratory populations [39].

Blue whale sightings in the Galapagos are year-round, though they are rare, with no more than 65 sightings reported over the last two decades [29,36]. However, they have recently increased due to citizen science programs [36,40]. This study aims to provide additional insights into the migratory behavior of blue whales in the Galapagos region, exploring whether their year-round presence is due to non-migratory residents or seasonal visitors from the NEP or SEP populations [36]. Satellite tags were deployed on *B. musculus* individuals in the Galapagos over three years. By analyzing the tracked movements of these whales alongside remote sensing data on oceanographic parameters, the study seeks to uncover factors driving their migration patterns and explore population interconnections. During the tagging years, we conducted an analysis of El Niño–Southern Oscillation (ENSO) conditions to provide an explanation for the presence of the whales in the archipelago. The findings are expected to inform conservation and management strategies by enhancing our understanding of how oceanographic conditions affect whale distribution and behavior within the Galapagos and the broader ETP.

## 2. Materials and Methods

### 2.1. Tagging Procedures

Sixteen blue whales were tagged in the Galapagos archipelago, ten in 2021, four in 2022, and two in 2023 (Table 1). A transdermal satellite transmitter model SPOT-372A manufactured by Wildlife Computers (Redmond, WA, USA) was used. The tag model used specifies a battery life of 540 days, assuming 250 Argos transmissions per day. Factory transmitters are 293 mm in length and 24 mm in diameter for the stainless-steel tube case and weigh 390 g, coupled to a stainless-steel spear with a 3 cm triangular double-edged blade tip containing three pairs of 5 cm barbs placed at 90° to each other.

To maximize battery life, transmitters were programmed to limit transmissions to a time block from 01:00 to 22:00 h every 2 days and to slow the repetition rate after 10 consecutive dry transmissions. Tags were chemically sterilized and plastic-wrapped in the laboratory. Before deployment, the tag/spear was sprayed with the antibiotic Neomycin Sulfate—Clostebol Acetato (Neobol^®^, Luminova Pharma Group, Guatemala City, Guatemala). The Animal Care and Use Committee of the Smithsonian Tropical Research Institute approved the procedure.

Whales were tagged from a long fiberglass-hull inflatable boat at approximately 2–4 m. Tags were deployed using an ARTS pneumatic line-thrower (Restech Inc., Bodø, Norway) coupled to a carrier/launching tube (Wildlife Computers, Redmond, WA, USA). A detailed description of the tagging procedure is provided elsewhere [41,42].

### 2.2. Track Correction and Environmental Covariates

The tag-derived positions from Argos satellite location classes “3”, “2”, “1”, “0”, “A”, and “B” were used with a range of errors in accuracy estimated to be between a 150 m and 5 km radius for plotting general filtered whale movements (see [43,44]). The location classes represent different levels of positional accuracy, where the standard quality signals of “3”, “2”, and “1” are derived from four or more messages and the low-quality locations are classes “0”, “A”, “B”, and “Z”, derived from three or two messages considered less accurate [45]. Raw transmissions were filtered to avoid unrealistic traveling speeds faster than 3 m s^−1^ [46], as well as transmissions occurring on land and with an Argos quality of “Z”. Density distribution within the insular exclusive economic zone of Ecuador, including the Galapagos Marine Reserve, was assessed using kernel density plots to understand whale habitat utilization. The analysis categorized areas into the following three tiers: low-use areas, representing regions from the smallest values (excluding 0) up to the first quartile (25% of the utilized habitat); intermediate-use areas, spanning from the first to the third quartiles (50% of the utilized habitat); and high-use areas, comprising values above the third quartile (representing 75% of the utilized habitat). For the kernel density estimation, the output cell size was manually set to 0.01 to improve the raster resolution, the search radius was automatically determined using the software to optimize the spatial resolution of the density estimation, and the EEZ (exclusive economic zone) was used as a boundary to ensure the analysis was constrained to the study area. Data analyses and visualization were conducted using the spatial analyst tool in ArcGIS Pro V3.2 (ESRI).

We used a state–space model to analyze whale movements to correct for errors derived from the Argos satellite tracking data. In addition, it allowed us to interpret the behavior of the whales at each individual location. We used movement persistence, an index of movement behavior that can be estimated as a continuous-valued variable (ranging from 0 to 1) which is time-varying, representing changes in the movement pattern based on autocorrelation in speed and direction [47]. We employed the “fit_ssm()” approach with the “model = mp” option to estimate move persistence accurately. This approach consists of fitting a continuous-time state–space model of motion persistence (MP), which allows us to infer the actual locations and the degree of motion persistence. We selected this method because it effectively handles the irregular timing and error-prone nature of telemetry data from the Argos satellite system while still capturing key behavioral patterns in whale movement [48]. We used the “fit_ssm” function from the aniMotum package [48] in R version 4.2.3 [49] with a speed filter threshold of 3 m s^−1^ [33] and a 6 h time step.

Estimated locations based on the state–space model results were analyzed with a hidden Markov model (HMM) to identify different behavioral states and correlate them to environmental variables. The HMM model was run using the function “fitHMM”, available in the R package moveHMM [50]. Initial values were set to 60 ± 10 km for migratory behavior, 20 ± 10 km for foraging behavior, and π to 0 for the turning angle, considering data availability and whale speed [33] (Table 2). Monthly chlorophyll-a concentration, 3-day composite productivity, and daily sea surface temperature were used as model covariates and were obtained from the NOAA’s Environmental Research Division ERDDAP server (NOAA ERDDAP, 2023). The temporal and spatial pairing between estimated locations and environmental data was performed using the R software “xtracto” function in the xtractomatic package [51]. To examine the possible correlations between the environmental variables, a correlation matrix was calculated using R software. A Wilcoxon rank-sum test was employed to investigate the relationship between chlorophyll (Chl), primary production (Prod), sea surface temperature (SST), and the different states of foraging and migration. This test was carried out with a significance threshold of 0.05 to determine if there was a statistically significant association between Chl, Prod, SST, and the foraging and migration states.

To describe the distribution as a function of depth, bathymetric data were obtained from the GEBCO dataset [52]. The bathymetric map was created in ArcGIS Pro v3.2, employing the shaded relief tool and overlaid with the hidden Markov model (HMM) result. To assess the statistical differences in depth between these two behaviors, a Wilcoxon rank-sum test was performed using R v4.2.3. Depth data ranges were divided into five arbitrary depth categories, and each modeled transmission was assigned to one of these ranges.

### 2.3. El Niño–Southern Oscillation

We used daily satellite data from the Copernicus Marine Service to assess the impact of the ENSO on critical oceanographic conditions in the Galapagos archipelago during our whale tagging expeditions [53,54,55,56]. These data spanned the period from 2020 to 2023 and included various oceanographic parameters essential to the study, such as chlorophyll concentration, primary productivity, and sea surface temperature. This analysis aimed to determine whether ENSO-related environmental changes, such as reduced chlorophyll and productivity, along with higher sea surface temperatures, may have contributed to the observed reduction in whale sightings during 2023.

The acquired satellite data underwent preprocessing steps to enhance their suitability for analysis. Initially, the raw daily data were filtered to reduce noise and variability by computing quarterly averages. This averaging process facilitated the extraction of seasonal trends and mitigated the effects of short-term fluctuations.

A rectangular zone extending 200 nautical miles from the outermost edges of the archipelago was delineated for analysis. The preprocessed satellite data for each year were imported into ArcGIS Pro version 3.2 for visualization and spatial analysis.

To further assess the ENSO phenomenon, the Oceanic Niño Index (ONI) was evaluated and visualized for the four-year period under study. The ONI data were sourced from the NOAA Climate Prediction Center, which provides information on warm and cold periods based on a threshold of ±0.5 °C for the ONI. The ONI is derived from the 3-month running mean of ERSSTv5 sea surface temperature (SST) anomalies in the Niño 3.4 region (5° N–5° S, 120°–170° W). The ONI for the period from 2020 to 2023 was plotted using the “ggplot2” package in R version 4.2.3.

## 3. Results

**Figure 1:**
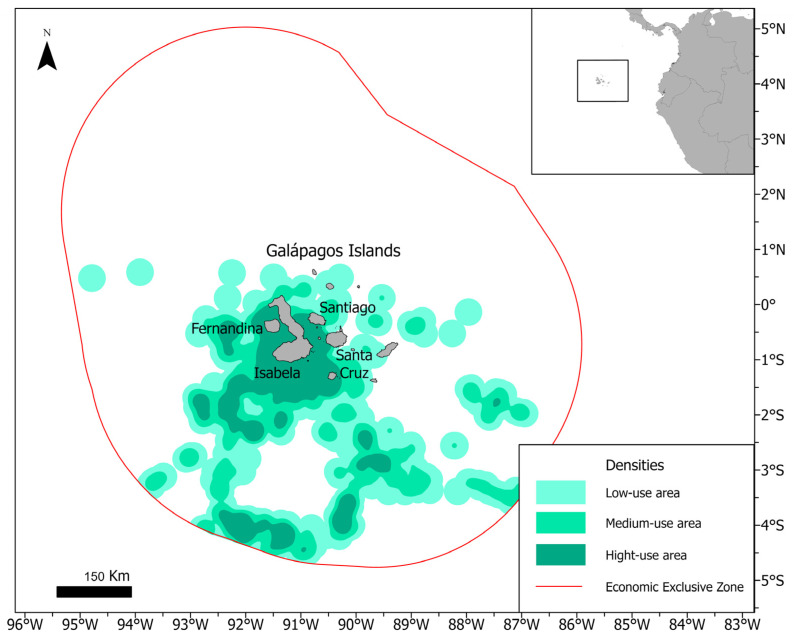
Kernel density of high-, medium-, and low-use areas used by blue whales tagged in the Galapagos Islands.

All blue whales were tagged during the austral winter season (May–October), specifically during the period with the highest number of sightings [36]. Sixteen blue whales made substantial journeys in various directions from their initial point of tagging during three different years, which were 2021, 2022, and 2023. The maximum distance covered was approximately 2400 km over a span of 98 days. This translates to an average speed of 25 km per day or 1 km per hour. The kernel density analysis identified regions of high-density usage around the Galapagos Islands and southwest of Ecuador’s exclusive economic zone (EEZ). Medium-density zones were observed in the southwestern vicinity of the islands, contiguous with the high-density areas in the south of the EEZ, while low-density regions were predominantly situated around the islands and in the southern expanse of the EEZ (Figure 1). On average, whales spent 22.98 ± 37.7 (54 maximum; 1.9 minimum) days inside the Galapagos’ EEZ (Table 1).

**Figure 2:**
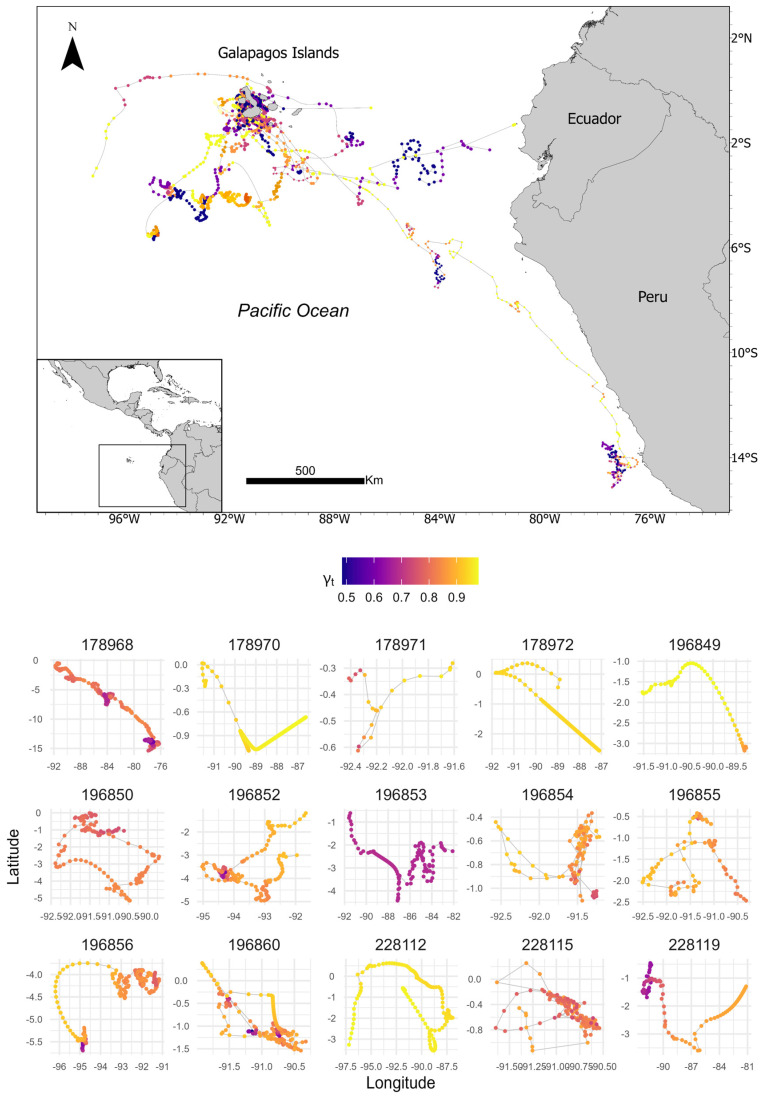
Blue whale migration using the state–space model, tagged in 2021, 2022, and 2023. Lower gamma_t values (ranging from orange to purple) highlight regions where the whale is likely engaged in area-restricted search behavior, while higher gamma_t values (shifting from light orange to yellow) indicate areas where whales exhibit directed, rapid movements. Numbers indicate the whale’s identity.

After track correction using the state–space model, successful modeling was achieved for 15 of the 16 whales, as whale ID 228114 could not be modeled due to less than 10 transmissions of good quality within 15 days. The initial database, which encompassed all whales, comprised 5990 Argos locations classified into error classes as follows (in descending order): B (62.68%), A (18.14%), 0 (7.18%), 1 (6.94%), 2 (2.74%), 3 (1.77%), and Z (0.55%). Following track correction of the state–space model, the database increased to 3448 coordinate locations (Figure 2).

### 3.1. Identification of Behavioral States and Their Relationship with Environmental Variables

**Figure 3:**
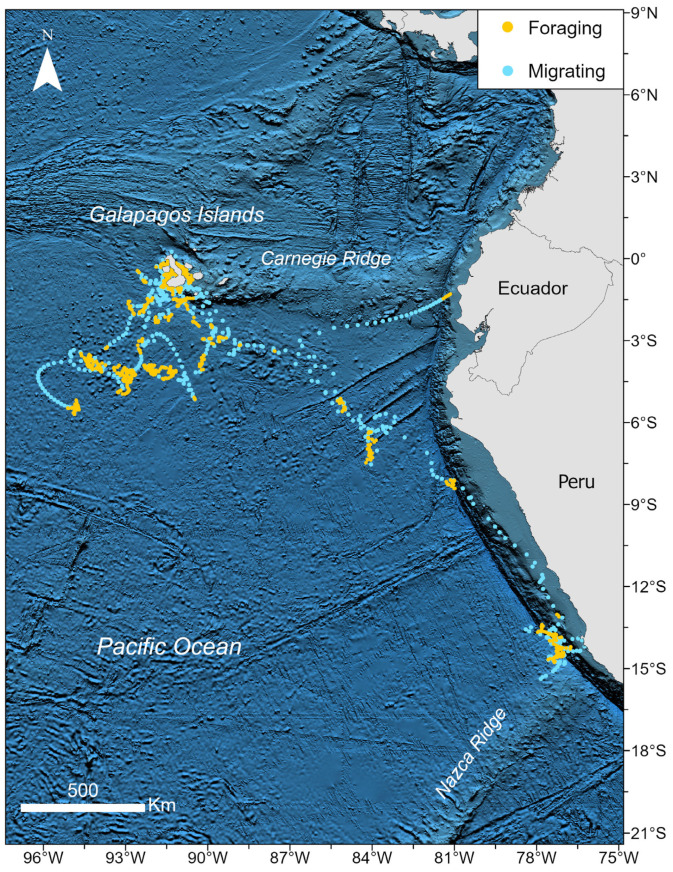
Migration (blue dots) and foraging (yellow dots) states of blue whales tagged in the Galapagos archipelago inferred by HMM modeling over GEBCO bathymetry [50].

The hidden Markov model (HMM) could be applied to eight (whales id: 178968, 196850, 196852, 196854, 196855, 196856, 228115, and 228119) of the sixteen whales originally tagged because of the extent and nature of the data, as many of these whales transmitted for longer periods. The model delineated two distinct behavioral states, namely migrating, characterized by a step distance (distance between time intervals) of 25.12 km (±15.16 km) and a turning angle of about 0.002 radians, and foraging, characterized by a step distance of 7.06 km (±5.07 km) and a turning angle of approximately −0.086 radians (Figure 3). The swimming patterns during foraging indicate an effort to remain within a prey patch, while migration travel behaviors involve movement between patches.

Whales used different zones for foraging based on bathymetry. It was observed that whales appeared to travel south from the Galapagos Islands along the continental shelf margin, creating a significant foraging hotspot at the convergence of the continental shelf with the Nazca Ridge. Additionally, whales seemed to travel adjacent to ridges, with a notable feeding area at the intersection of the Carnegie Ridge and the continental shelf. This pattern indicated several feeding zones around the Galapagos Islands, the Nazca Ridge, and other regions, highlighting a strong link between bathymetric features and foraging behavior (Figure 3). Statistical analysis confirms significant differences in depth between feeding and migrating states (W = 204,191, *p* < 0.05). Whales tended to feed in deeper waters compared to when they were migrating. Specifically, 82.30% of the foraging locations were in waters deeper than 1500 m, whereas only 70.55% of the locations during migration were in similarly deep waters (Table 3).

**Figure 4:**
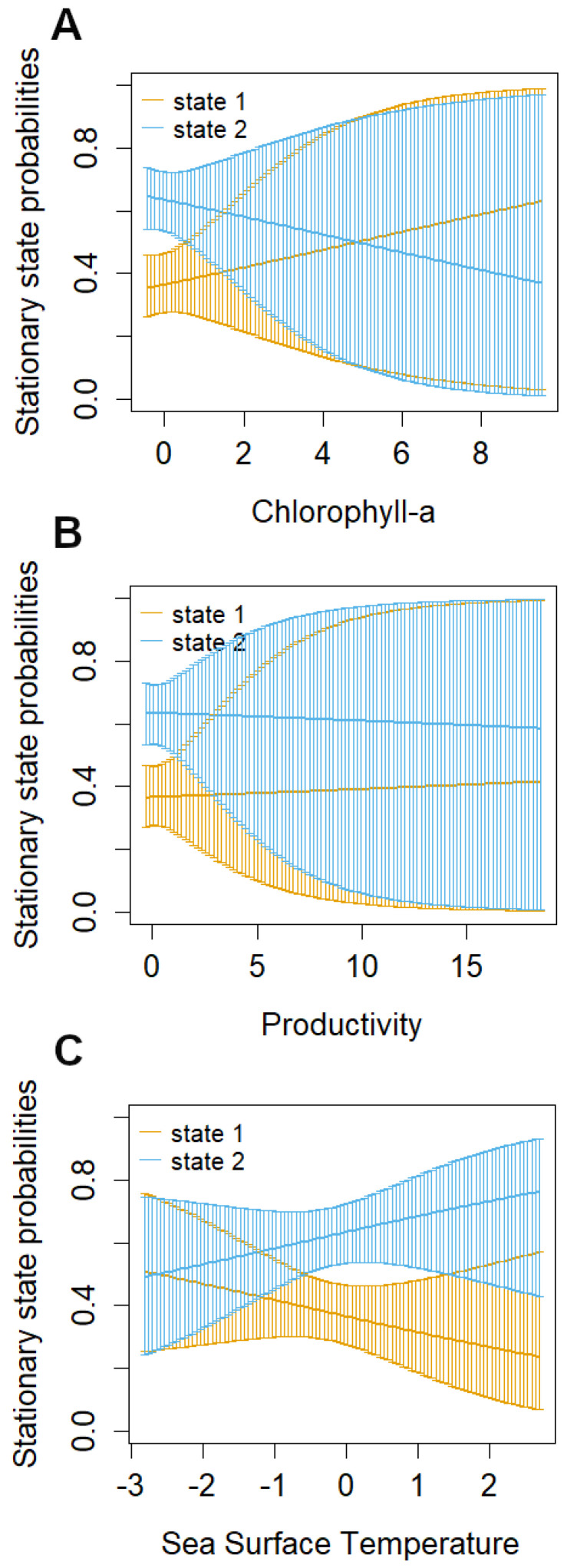
Long-term probabilities of blue whales at different values of the covariates. Chlorophyll (**A**), productivity (**B**), and sea surface temperature (**C**) in each behavioral state, foraging (orange: state 1) and migrating (blue: state 2), with alpha = 0.9 confidence intervals.

The likelihood of transitioning between these behavioral states (from migrating to foraging or vice versa) exhibited correlations with local environmental conditions under stationary long-term distribution (i.e., a stable pattern of behavioral states over an extended period) (Table 4). Specifically, the probability of a whale being in a foraging state increased as chlorophyll and primary productivity rose and decreased as sea surface temperature levels rose, while the probability of being in a migratory state decreased with increasing Prod and Chl, and increased with increasing SST (Figure 4A–C).

**Figure 5:**
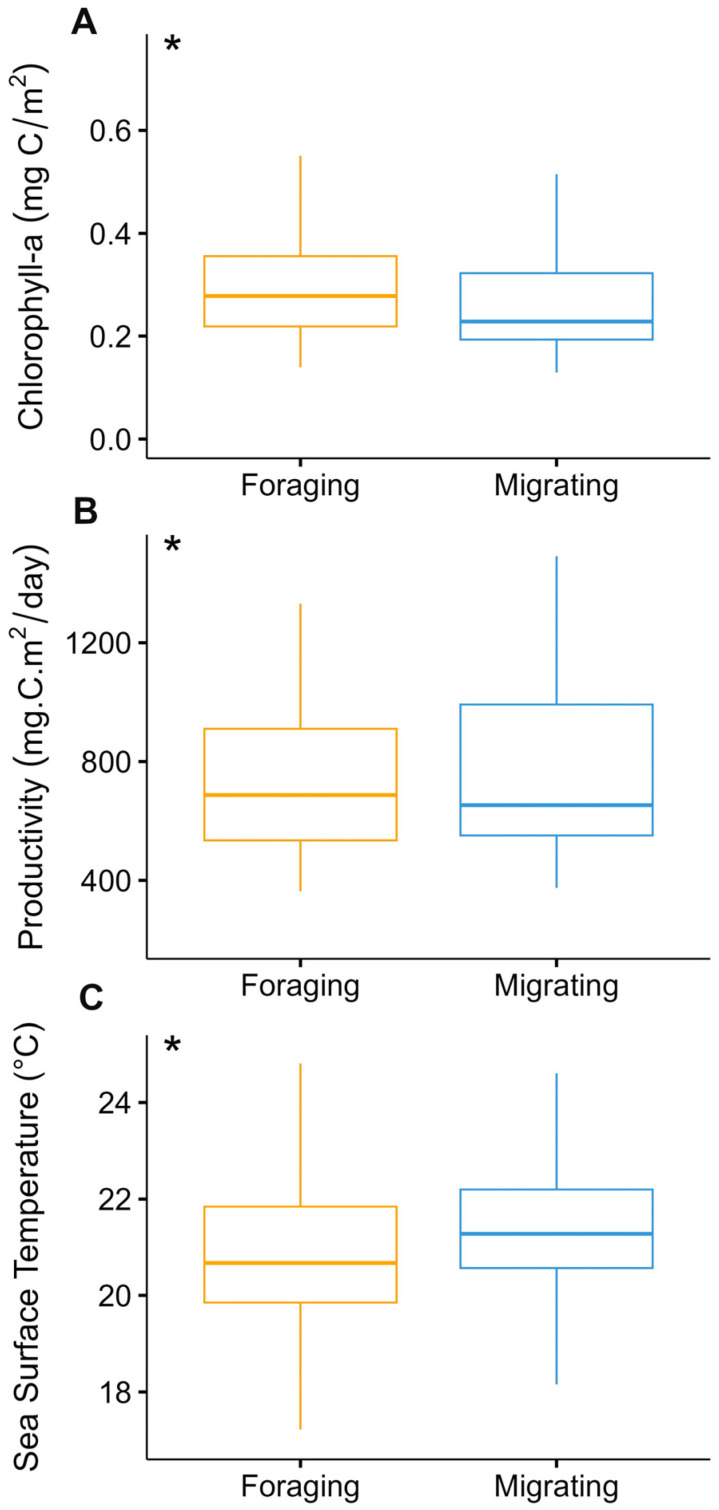
Environmental conditions during migration and foraging states modeled by a hidden Markov model. Blue whales preferred to forage when chlorophyll and primary productivity levels were higher and when sea surface temperature was lower. Stars indicate a significant difference.

The correlation matrix for environmental variables showed that there were no relationships between them. Tracked whales predominantly occupied waters with Chl levels averaging 0.81 ± 1.53 mg m^−3^, a Prod of 1410.96 ± 2892.40 mg C m^2^ day^−1^, and a SST of 21.69 ± 1.59 °C. There was a significant contrast in Chl, Prod, and SST levels between behavioral states. When blue whales were foraging, the Chl levels were notably higher (0.97 mg m^−3^) compared to when they were in the migrating state (0.72 mg m^−3^) (*W* = 250,233, *p* < 0.05, Figure 5A). In the case of Prod, blue whales demonstrated a preference for areas with higher productivity, recording 1839.55 mg C m^2^ day^−1^ during foraging compared to 1201.48 mg C m^2^ day^−1^ during migratory behavior (*W* = 54,912, *p* < 0.05, Figure 5B). Sea surface temperature (SST) differed significantly between the two behavioral states. During migration, the average SST was 21.8 °C, while during foraging, it was 21.5 °C (*W* = 187,499, *p* < 0.05, Figure 5C).

### 3.2. El Niño–Southern Oscillation

**Figure 6:**
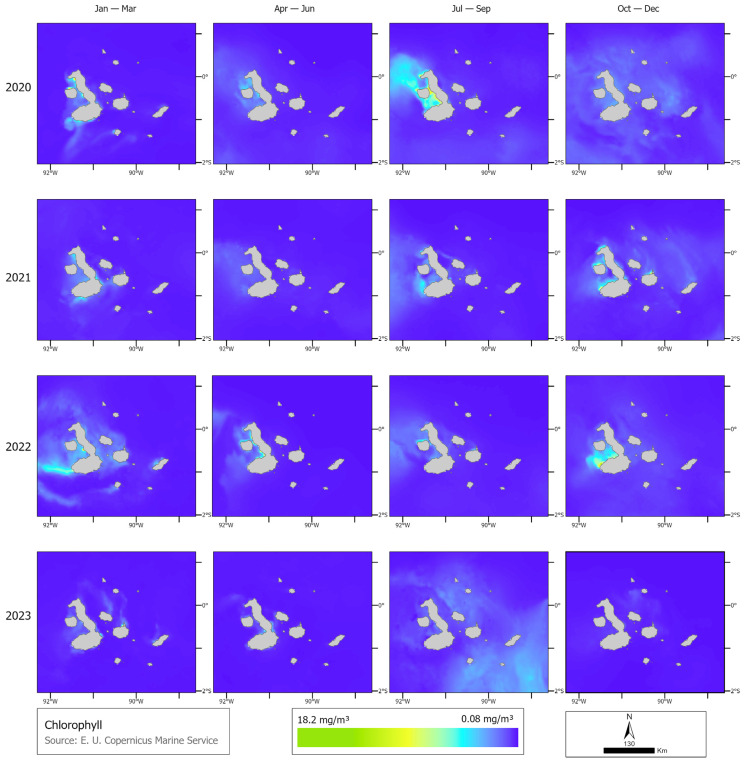
Difference in quarterly chlorophyll concentration (mg m^−3^) per year (2020, 2021, 2022, and 2023).

**Figure 7:**
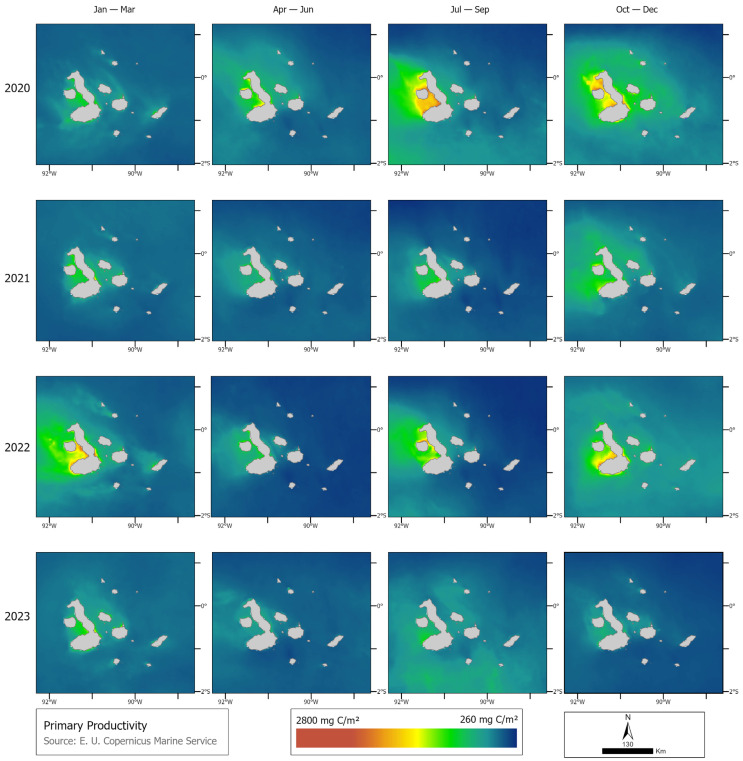
Difference in quarterly primary productivity (mg C m^−2^) per year (2020, 2021, 2022, and 2023).

Fewer whales were observed and consequently tagged in 2023 during the El Niño event, even considering that the yearly field effort was nearly three times that of the previous two expeditions. Chlorophyll in 2023 was lower than in previous years (2020, 2021, and 2022). In 2020, the maximum chlorophyll peak occurred between July and September, with a maximum of 13.18 mg m^−3^, and the period with the lowest chlorophyll was between October and December, with a maximum of 1.85 mg m^−3^. In contrast, in 2021, the highest chlorophyll concentration occurred from October to December, reaching 10.16 mg m^−3^, while the lowest concentration was 0.97 mg m^−3^ between April and June. In 2022, the highest chlorophyll levels were also from October to December, with a maximum of 18.26 mg m^−3^, while the lowest levels were recorded between July and September, at 5.78 mg m^−3^. Finally, in 2023, the highest chlorophyll concentration was 8.45 mg m^−3^ between January and March, and the lowest was between October and December, with a maximum of 0.83 mg m^−3^ (Figure 6). Coinciding with chlorophyll, primary productivity was lower in 2023 compared to the previous three years. In 2020, the maximum primary productivity was between July and September, with 2809 mg C m^−2^, and the minimum was between January and March, with a 1466 mg C m^−2^ peak. In 2021, the maximum was in the first four-month period of the year, with a maximum of 2122 mg C m^−2^, and the period with the lowest primary productivity was between July and September, with a maximum of 1484 mg C m^−2^. In 2022, the months with the highest primary productivity were between July and September, with a maximum of 2797 mg C m^−2^, and the minimum was between January and March. Finally, in 2023, the maximum was between January and March, which peaked at 1831 mg C m^−2^, while the minimum was between April and June, with a maximum of 1185 mg C m^−2^ (Figure 7).

**Figure 8:**
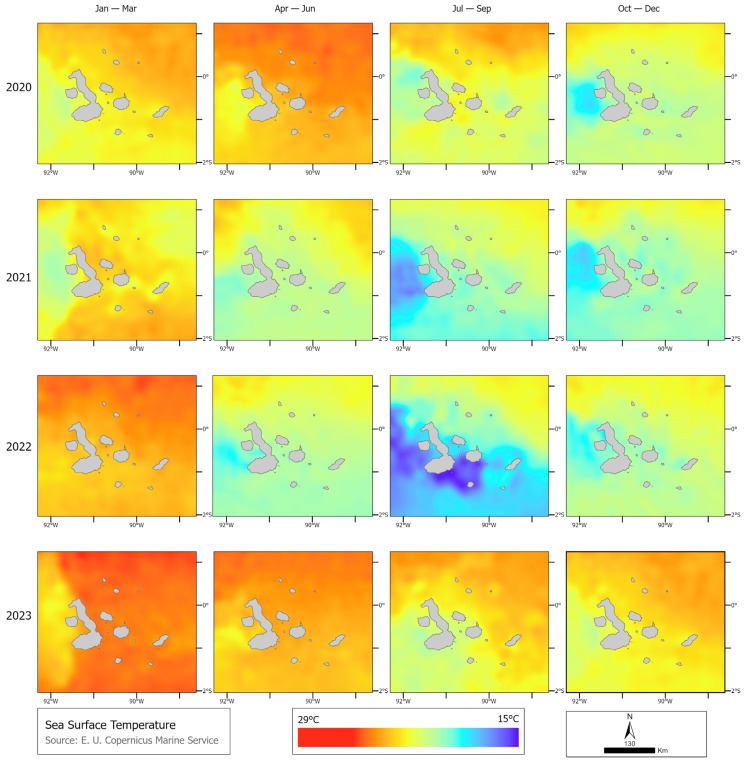
Difference in sea surface temperature (°C) per year (2020, 2021, 2022, and 2023).

Regarding SST, it was observed that 2023 was warmer than previous years. The coldest months were July to September 2021 and July to September 2022, whereas in 2023, it was considerably warmer across the years, reaching a peak of 29 °C. In 2020, sea surface temperatures (SST) peaked from April to June at 29 °C, with the lowest recorded in July to September at 21 °C. In 2021, the highest temperature occurred between January and March, reaching 27 °C, while the lowest was between July and September, ranging from 25 °C to 17 °C. Similarly, in 2022, the peak was from January to March at 27 °C, and the lowest temperature occurred between July and September, ranging from 25 °C to 15 °C. Notably, temperatures soared in 2023, peaking at 29 °C between January and March and April to June, with the lowest temperature observed from July to September at 22 °C, surpassing any values recorded in the previous four years (Figure 8).

**Figure 9:**
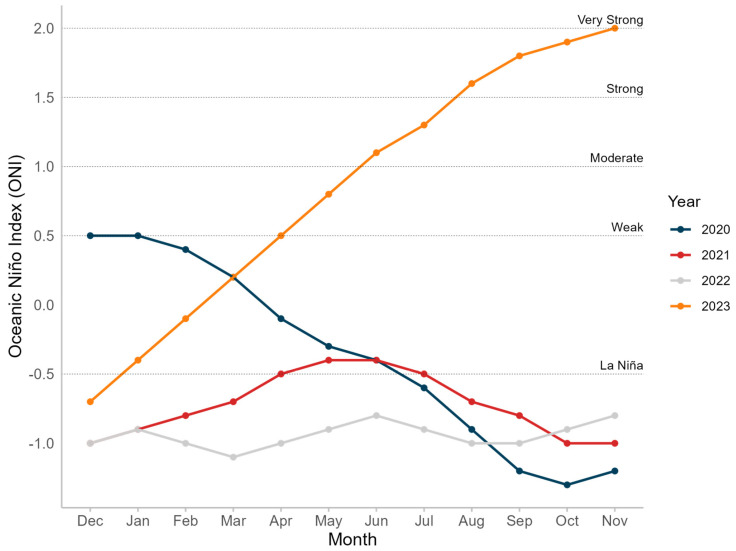
Oceanic Niño Index (ONI) from 2020 to 2023.

Additionally, the ONI data indicated that during the period from July to August 2023, the ONI was at a moderate level. However, from September to November 2023, the ONI was strong, reaching very strong levels in November. For the remaining years, the ONI showed La Niña conditions, except for 2020, where the early months exhibited weak El Niño conditions (Figure 9).

## 4. Discussion

This study aimed to elucidate blue whales’ poorly understood movement patterns in the Galapagos archipelago and beyond. It is the first to employ satellite tagging of blue whales within the Galapagos. By integrating data from these tags with the remote sensing of productivity proxies, we sought to understand the influence of these factors on their migratory and foraging behavior. It was previously believed that blue whales use the Galapagos during the cooler seasons (austral winter and spring) for breeding and return to their southern feeding grounds off Chile and Peru during the austral summer [31,57]. However, occasional sightings of blue whales in the Galapagos during the warmer season indicated a year-round presence [36,58]. Our findings indicate that most tagged whales remained near the islands for the duration of the transmission period. However, due to the temporal constraints of the transmission data, we could not definitively confirm continuous year-round residency. Nevertheless, our data underscore the significance of the Galapagos archipelago as a critical foraging area for these blue whales.

The Galapagos Islands are renowned for their marine mammal diversity, particularly cetaceans, with 23 species documented, some of which are residents [59]. Historically, the Galapagos archipelago was a major hotspot for whaling in the late 18th and 19th centuries [31,50,51,52,53,54,55,56,57,58], further emphasizing its longstanding significance as a breeding and foraging ground for resident and migratory whales. The region’s unique hydrographic conditions create a transition zone between tropical and subtropical waters, characterized by strong local and seasonal upwelling [60]. This increases primary productivity, particularly in the western part [60,61], leading to higher cetacean densities in this area [62]. Our results corroborate this, showing that whales predominantly use the western part of the archipelago, yet with the highest occurrences around Isabela Island rather than the previously described Canal Bolivar (Figure 1) [36,58]. This information is crucial for managing tourist operations to minimize disturbances to the animals’ feeding and calving behaviors and to mitigate the risk of ship strikes.

While most individuals remained close to the Galapagos, two foraged in the Ecuador EEZ and one (#178968) off Peru, indicating linkages to the SEP, as previously shown [23,29,30,38,39]. However, recent evidence from Guzman and Estevez [39] indicates that the Galapagos may also serve as a stepping stone, connecting northern and southern Pacific blue whale populations.

Overall, blue whale migratory routes have been shown to align with the trajectories of dense krill aggregations [54,55,56,63,64]. Long-term memory likely facilitates the annual return of blue whales to foraging grounds, where food availability remains consistently high over extended periods [63]. Submarine geomorphic features such as ridges, canyons, seamounts, and oceanic islands may also serve as navigation cues for baleen whales [65,66,67,68]. These features influence hydrographic conditions, resulting in local upwelling and changes in nutrient and SST (e.g., El Niño) levels supporting substantial krill populations [28,55,56,69,70]. Moreover, they could serve as potential navigational landmarks during long-distance migrations, though this aspect remains inadequately understood [67,71,72].

We observed associations between geomorphic features and foraging behaviors, including migrations along continental shelf edges and ridges, i.e., the Carnegie and Nazca ridges, previously identified as critical migratory routes for blue whales; the Salas y Gómez and Nazca ridges are recognized as regular migratory pathways for the Chilean blue whale en route to the Galapagos Islands for feeding [23,73,74,75]. The Carnegie Ridge has been recently declared an Important Marine Mammal Area of 459,869 km^2^ (https://www.marinemammalhabitat.org/factsheets/carnegie-ridge-galapagos-to-mainland-imma/ [accessed on 20 June 2024]). Whales in our study utilized the Nazca and Carnegie ridges for feeding, likely due to the high productivity in these regions. Northern Peru benefits from the nutrient influx driven by the northern Humboldt Current system [76] and the Nazca Ridge exhibits increased biological productivity [77]. Meanwhile, Carnegie Ridge, a transition area between the Humboldt Current and the eastern Pacific equatorial front, exhibits high surface productivity due to divergent equatorial upwelling and the advection of cool, nutrient-rich waters [78].

Blue whales show a distinct preference for feeding in various bathymetric zones, notably favoring deeper waters. This depth preference is likely a reflection of the role bathymetric features play in concentrating planktonic organisms, which attract blue whales to these highly productive areas [79,80]. Nevertheless, this does not necessarily mean that the whales are feeding at greater depths directly; rather, it suggests an association with regions where bathymetric conditions enhance overall productivity. Thus, while blue whale feeding behavior is linked to deeper waters, it is the increased productivity facilitated by these bathymetric features that likely drives their foraging preferences.

Our study established a link between environmental correlates—specifically sea surface temperature (SST), chlorophyll-a (Chl-a) concentrations, and productivity—and the movement behavior of blue whales. Foraging activity was notably more prevalent in regions with lower SST and higher Chl-a concentrations and productivity, indicative of high prey densities (e.g., [53,54,55,81]). This correlation suggests a feedback loop, where productive foraging areas also exhibit high whale densities, reinforcing the importance of these regions as critical habitats for blue whales. This relationship is important as it implies that environmental correlates can be utilized to predict areas of probable whale occurrence, thereby aiding in identifying critical habitats essential for managing and conserving endangered species [82].

Blue whales also demonstrate the ability to adapt temporally [83,84] and spatially [64] to their prey’s patchy and ephemeral nature. For example, Szesciorka et al. [83] discovered that seasonal variations in SST in the California Current Ecosystem led to blue whales arriving earlier for feeding. Cooler temperatures were associated with higher krill biomass in the following season, demonstrating the whales’ ability to adjust their migratory timing in response to interannual environmental changes. SST, therefore, acts as a migratory cue influencing their behavior [53,54,55]. Understanding these patterns is key for conservation, particularly in timing the whales’ arrival at feeding and breeding grounds, which can inform temporal conservation measures [83]. Conducted during a strong ENSO year (2023), our study identified substantial changes in SST and surface primary production. Despite the lack of discernible behavioral differences—likely due to the limited sample size of only two tagged whales in 2023—the environmental perturbations driven by the ENSO could significantly impact resource availability. Conversely, the limited number of specimens tagged, despite intensified efforts this year, suggests potential changes in whale presence or behavior during significant ENSO events. As more frequent and extreme ENSO events are predicted (e.g., [85]), these changes can impact blue whale behavior, habitat utilization, and reproductive success [83,86].

## 5. Conclusions

Our findings highlight the importance of understanding blue whale migratory and foraging patterns to inform conservation strategies. The alignment of blue whale migratory routes with high-productivity areas underscores the necessity of protecting key foraging grounds that provide consistent food resources. As environmental changes, such as ENSO events alter chlorophyll, primary productivity, and SST, blue whales may have to adapt their migratory and foraging behaviors. This adaptability suggests a need for dynamic conservation measures that can respond to shifting environmental conditions.

Additionally, heavy ship traffic along migratory routes, particularly the presence of tourist vessels in areas of high whale density like Isla Isabela in the Galapagos [87], may pose a significant threat to blue whales (see also [88]). Implementing measures to mitigate ship strikes, such as limiting the number of boats or their proximity to whales, altering shipping lanes, and enforcing speed restrictions in critical habitats, is essential to reduce mortality risks (e.g., [89,90]). Conservation efforts must also account for the impacts of climate change, which could further affect the availability and distribution of prey, necessitating a comprehensive approach to ensure the long-term survival of blue whale populations. By integrating environmental correlates into conservation planning, we can better predict whale occurrence and protect essential habitats [33,82]. Continued research and monitoring are vital to adapting conservation strategies to the dynamic nature of marine ecosystems, ultimately supporting the preservation of these magnificent ocean giants.

## Figures and Tables

**Table 1:** Summary of tracked blue whales tagged off the Galapagos Islands and their time within Ecuador’s economic exclusive zone (EEZ).

Id	Tagging Date (mm-dd-yyyy)	Last Date (mm-dd-yyyy)	Transmissions (Days)	Traveled Distance (km)	EEZ (Days)
178968	10-02-2021	08-01-2022	98.13	11,395.47	23.08
178970	10-03-2021	08-30-2022	331.91	4341.43	4.04
178972	08-03-2021	09-27-2021	54.55	1045.77	54.55
196849	10-07-2021	10-20-2021	13.59	4262.14	13.59
196850	10-04-2021	11-08-2021	34.99	3471.42	33.88
196852	10-04-2021	11-15-2021	42.15	3228.02	12.04
196853	10-02-2021	10-29-2021	27.49	3324.29	13.02
196854	10-05-2021	11-03-2021	28.82	3138.75	28.82
196855	10-02-2021	10-27-2021	24.52	2004.12	24.52
196856	11-06-2021	01-01-2022	56.00	4026.65	26.32
178971	09-26-2022	10-01-2022	5.15	2369.53	5.15
196860	10-06-2022	11-18-2022	43.00	3139.70	43.00
228112	10-01-2022	11-04-2022	34.45	3076.18	28.83
228114	10-14-2022	10-29-2022	14.49	1424.57	1.94
228115	09-27-2023	11-03-2023	37.51	2508.08	37.51
228119	09-23-2023	10-17-2023	23.77	2675.84	17.4

**Table 2:** Parameter estimates for behavioral states in the blue whale hidden Markov model.

Model Parameter	Behavioral State	Value
Step Length (km)	Migratory	60 ± 10
	Foraging	20 ± 10
Turning Angle	Migratory	0
	Foraging	π

**Table 3:** Percentage of whales’ distribution area with respect to depth (m) and states (foraging and migrating) as estimated by the HMM model.

Depth (m)	Foraging (%)	Migrating (%)
0–200	6.44	8.14
201–500	2.21	8.35
501–1000	5.63	6.59
1001–1500	3.42	6.37
>1500	82.30	70.55

**Table 4:** Model coefficient of correlation between each environmental variable (HMM model covariate) to the probabilities of switching between behavioral states (foraging and migration), with confidence intervals.

	From Foraging to Migrating State	From Migrating to Foraging State
Intercept	−1.957 (−2.354, −1.560)	−2.569 (−2.963, −2.174)
Chl	−0.069 (−0.415, 0.276)	0.057 (−0.347, 0.460)
Prod	0.062 (−0.166, 0.290)	0.070 (−0.228, 0.368)
SST	−0.174 (−0.545, 0.197)	−0.405 (−0.895, 0.085)

## Data Availability

The data supporting this study’s findings are available from the first author [HMG] upon reasonable request due to privacy restrictions.
